# The Use of Venous Catheter and Irrigation with Povidone-Iodine 0.6% in Patients with Punctal and Proximal Canalicular Stenosis: Preliminary Report

**DOI:** 10.3390/jcm13051330

**Published:** 2024-02-26

**Authors:** Claudia Azzaro, Alessandro Meduri, Giovanni William Oliverio, Laura De Luca, Francesco Gazia, Francesco Franchina, Pasquale Aragona

**Affiliations:** 1 Bioscenses Departement, Ophthalmology Clinic, University of Messina, 98124 Messina, Italy; claudiaazzarorg@gmail.com (C.A.); ameduri@unime.it (A.M.); giovannioliverio89@gmail.com (G.W.O.); franchina.francesco8@gmail.com (F.F.); paragona@unime.it (P.A.); 2 Unit of Otorhinolaryngology, Papardo Hospital, AO Papardo C. da Papardo 1, 98158 Messina, Italy

**Keywords:** obstruction of lacrimal drainage system, venous catheter, povidone-iodine, NIKBUT, TMH

## Abstract

**Background:** This study aimed to evaluate the safety and efficacy of povidone-iodine 0.6% (PVI) irrigation for preventing recurrence of stenosis after punctoplasty in patients with punctal and proximal canalicular stenosis treated using a venous catheter as a stent. **Methods:** Twenty patients were enrolled and divided into two groups. Group 1 received irrigation of 1 mL 0.6% PVI, while Group 2 received 1 mL of balanced salt solution (BSS). The patients underwent baseline, 15-, 30-, and 90-day assessments using the Ocular Surface Disease Index (OSDI) questionnaire, Symptoms Assessment in Dry Eye (SANDE), Schirmer I test, tear meniscus height (TMH), bulbar redness, meibography, and non-invasive breakup time (NIKBUT) through Keratograph 5M (Oculus, Germany). **Results:** At three months, both groups demonstrated statistically significant improvements in symptoms and ocular surface parameters. However, Group 1 showed statistically significant improvements in OSDI, SANDE scores, bulbar redness, and NIKBUT compared to Group 2. Additionally, no patients in Group 1 presented a recurrence of stenosis, while three patients in Group 2 demonstrated stenosis relapse at the end of the follow-up period. **Conclusions:** The application of a venous catheter and PVI 0.6% irrigations proved to be effective in treating proximal lacrimal duct stenosis, reducing the risk of recurrence and improving tear film stability, ocular discomfort symptoms, and ocular surface parameters.

## 1. Introduction

The lacrimal drainage system is composed of proximal and distal parts: the upper part consists of lacrimal puncta, inferior and superior canaliculi, and the common duct, while the lower part includes the lacrimal sac and the nasolacrimal duct. In the literature, there is no consensus on the incidence percentage of causes leading to alterations in the drainage of the lacrimal ducts. What is emphasized is that among stenosis/obstruction of the superior part, punctal stenosis is the most common, with an incidence ranging from 8% to 54.3% [1,2,3]. Lacrimal obstructions can be categorized as congenital or acquired, with the latter being more prevalent.

Clinically, patients with an obstruction at any point along the drainage system present with epiphora, which is the overflow of tears at the eyelid margin, and mucopurulent discharge [4]. The most commonly used grading system to evaluate epiphora is the Munk scale, consisting of six grades, ranging from zero (without epiphora) to five (constant epiphora) [5].

A punctal stenosis is typically diagnosed during the visit through slit lamp examination, and this parameter is crucial for assessing the severity of inflammation and fibrosis to determine the most appropriate treatment. In 2003, Kashkouli et al. introduced a lacrimal punctal score system (grades 0–5) [6,7].

To assess canalicular stenosis clinically, lacrimal irrigation can be employed. Radiologically, it can also be demonstrated through dacryocystography (DCG), magnetic resonance dacryocystography (MR-DCG), or computed tomography dacryocystography (CT-DCG) [8,9,10].

Various procedures have been proposed by different authors to treat acquired punctal and canalicular stenosis [11]. Surgical techniques include simple punctal dilation, punctal punching, insertion of a punctal plug, various punctoplasty techniques, the insertion of a toughened glass (Lester Jones) tube, external approach dacryocystorhinostomy (DCR), and stent application [5,6,7,8,9,10]. In recent studies, our group proposed a novel technique, inserting a vialon cannula from the inferior canaliculus to the lacrimal sac, used as a stent, with the advantage that it can be used for tear duct irrigation [10,11].

However, recurrence of stenosis is a significant post-operative complication of these treatments [3]. Tear stasis allows the persistence of cytokines and metalloproteinases, which could increase inflammation of the ocular surface and promote damage to epithelial cells and apoptosis [12,13]. These conditions lead to fibrosis of the eyelid margin, including puncta, and could be the basis for the recurrence of stenosis after surgical treatment [12,14]. Moreover, an increased load of microbial flora on the lid margin is correlated with increased inflammation on the ocular surface [15]. Ocular conjunctival flora comprises a wide range of bacteria that do not cause infection in normal conditions but can be a major contamination route during ocular surgery [16].

Recent studies have demonstrated the efficacy of 0.6% povidone-iodine (PVI) in treating ocular surface diseases, improving signs and symptoms in patients with dry eye disease (DED) and Meibomian gland dysfunction (MGD) [17]. PVI is an antiseptic with several properties, such as immediate broad-spectrum activity against different types of bacteria, with no reported microbial resistance. Moreover, PVI presents effective anti-inflammatory activity as well as great efficacy in wound healing. It has been shown to be safe and very well tolerated [17]. Although PVI is generally safe, some complications have been described in the literature, such as allergic reactions, ocular irritation, post-operative eye pain, and corneal toxicity [18,19].

Several studies have reported that low concentrations of PVI 0.6% present a much more effective bactericidal effect while still preserving the microbiota of the ocular surface [20]. Several in vitro studies have proven the antimicrobial efficacy of ophthalmic solutions containing 0.6% PVI, which showed a faster bactericidal activity compared to traditional 5% PVI solution [20].

In this study, we focused on superior section obstructions, aiming to describe a simple treatment technique consisting of the insertion of a venous catheter and to compare the effectiveness of irrigation with PVI 0.6% versus balanced saline solution (BSS). The goal of this study is to evaluate the efficacy of PVI 0.6% irrigation in patients with punctal and canalicular stenosis treated using a vialon cannula, to prevent the recurrence of stenosis and improve ocular surface parameters.

## 2. Materials and Methods

In this prospective study, 20 patients (11 males, 9 females) with punctal and proximal canalicular stenosis were enrolled and treated at the Ophthalmology clinic of the University of Messina. The study protocol was approved by the local institutional review board of the University of Messina and followed the tenets of the Helsinki declaration. Informed consent was obtained from all the patients enrolled in the study after a discussion of the potential risks and benefits and the nature of this study. The study included patients with chronic epiphora and punctal stenosis and/or proximal canalicular stenosis demonstrated by CT-DCG. Exclusion criteria included eyelid alterations such as ectropion or entropion, lower canalicular stenosis, canalicular obstruction, previous lacrimal system trauma, systemic or topical chemotherapy, regional radiation, radioactive iodine, conjunctivitis, known conjunctival inflammation, graft-versus-host disease, and congenital lacrimal agenesis.

Diagnosis of punctal stenosis was obtained through the dye disappearance test (considered positive if dye remains after 5–10 min), slit-lamp examination (external puncta size < 0.2 mm), and the incapacity of the 27-gauge cannula to pass through the puncta without dilation [12,13]. To evaluate canalicular patency, a size #00 Bowman probe was used: soft resistance to the probe indicates a canalicular system obstruction, while a hard stop suggests a patent canaliculus [14]. Distal obstruction of the lacrimal duct was excluded by lacrimal irrigation with 2 mL saline fluid.

**Figure 1:**
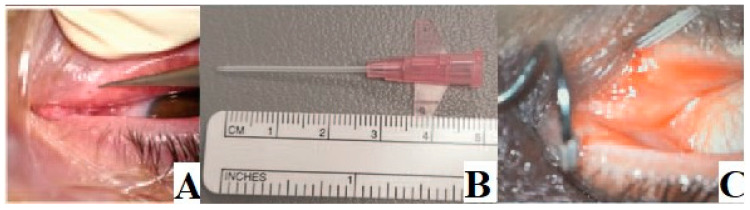
(**A**) Punctum dilatation; (**B**) Venous catheter as a stent; (**C**) Placement of the catheter in the inferior canaliculus through a pigtail and released to the superior punctal duct.

**Figure 2:**
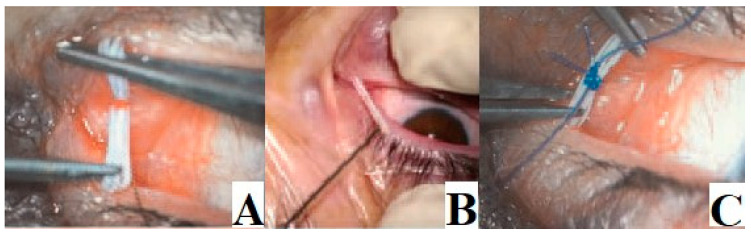
(**A**,**B**) The two ends of the venous catheter are placed one inside the other; (**C**) A safety suture with 4-0 nylon is performed, and the catheter is rotated to hide the safety suture inside the tear ducts and prevent rubbing against the cornea or conjunctiva.

Patients were treated using a venous catheter as a stent for punctal and proximal canalicular stenosis, and after 15 days, the implant was removed (Figure 1 and Figure 2). The operation was considered successful if the lacrimal way was canalized and there was no recurrence in the three-month follow-up period.

Furthermore, patients were randomly divided into two groups: group 1 (10 patients, 4 males, and 6 females) underwent canalicular irrigation of 1 mL povidone-iodine 0.6% (IODIM^TM^ ocular drops on preservative-free 0.6% PI, hyaluronic acid, and medium chain triglycerides, Medivis, Catania, Italy) on days 1, 3, 7, and 14, whereas group 2 (10 patients, 7 males, and 3 females) received irrigation of 1 mL of BSS.

### 2.1. Tests That Patients Performed at Baseline, 14, 30, and 90 Days

1. Ocular Surface Disease Index (OSDI) questionnaire: This questionnaire comprises twelve questions grouped into three categories or subscales (ocular discomfort, vision-related functionality, and environmental factors). A subject is classified as symptomatic when the total OSDI score is equal to or over thirteen points, with all twelve items carrying the same weight. All the patients completed a self-administered Italian version of the OSDI questionnaire. The values provided by the patients for each question and the sum of the values scored for the questions of each subscale were recorded. Patients with an OSDI score ≥13 were classified as symptomatic, in line with previous reports in the literature [21].
2. Symptoms Assessment in Dry Eye (SANDE) quantified dry eye symptoms by combining two visual analog scales that separately assessed the frequency and severity of dry eye symptoms. Each index used a 100 mm line to individually assess both the average frequency and the average severity of symptoms of ocular discomfort or dryness experienced by the patient [22].
3. Slit-lamp examination;
4. Schirmer I test was conducted to test basal and reflex tear secretion using a specialized Schirmer’s strip prepared from Whatman filter paper no. 41 measuring 40.5 mm, marked 0 to 35 mm without topic anesthesia. Depending on the wetting of the strip, the results of Schirmer’s test were graded as follows: >10 mm, normal (grade 0); 5–10 mm, mild (grade 1); 3–4 mm, moderate (grade 2); 0–2 mm, severe (grade 3). Filter paper strips (Test di Schirmer, Alfa Intes, Casoria, Italy) were applied at the junction between the outer and the middle third of the lower lid. The moisturized length of the strip was measured after 5 min (mm/5 min).
5. Tear meniscus height (TMH): TMH can be viewed under cobalt blue light using a slit lamp and fluorescein instillation; we took three consecutive measurements and considered as significant the mean value through the Keratograph 5M topographer (Oculus Optikgeräte GmbH, Wetzlar, Germany).
6. Conjunctival Bulbar Redness (CBR) through the Keratograph 5M topographer (Ocu-lusOptikgeräte GmbH, Wetzlar, Germany): This keratometer possesses a color camera optimized for external imaging (R-Scan module); it is able to measure ocular redness. This was carried out by a Placido ring system which scanned the exposed bulbar and limbar conjunctiva and immediately generates an image of 1156 × 873 pixels and five redness scores (RSs) on the computer screen. These RSs were continuous variables based on the percentage area ratio between the blood vessels and the rest of the analyzed area [23].
7. Meibography assessment through Keratograph 5M (Oculus, Germany): Meibography was performed in both upper and lower eyelid to evaluate the Meibomian gland dropout considering grade 0 (no glands’ loss), grade 1 (area of loss smaller than 1/3), grade 2 (area of loss between 1/3 and 2/3), and grade 3 (area of glands’ loss greater than 2/3), using the automatic assessment function of the Keratograph 5M (JENVIS Meibo Grading Scale). The MG area ratio, diameter deformation, tortuosity, and signal intensity are considered in the literature as promising biomarkers for MGD diagnosis and objective grading.
8. Non-invasive Keratograph break up time assessment through Keratograph 5M (Oculus, Germany) (NIKBUT): NIKBUT was measured by the automated capture of Placido disk images that are taken from the anterior ocular surface thanks to a corneal topography system. Computer software was then used to identify irregularities and the breakup of the precorneal tear film; therefore, the system was able to calculate the first and average time.

### 2.2. Statistical Analysis

The numerical data are expressed as mean and standard deviation, whereas the categorical variables as absolute frequency and percentage. The fitting of the data to a normal distribution was tested by the Kolmogorov-Smirnov test. The pre-operative and post-operative values of the variables of Group 1 and Group 2 were compared using the Chi-Square test for categorical variables, the Student *t*-test for parametric data, and the Mann-Whitney U test for non-parametric data. The post-operative outcomes were compared with pre-operative values within each group using the Wilcoxon signed-rank test. A *p*-value inferior or equal to 0.05 was considered significant.

## 3. Results

The clinical characteristics of the study populations are reported in Table 1; no statistically significant difference emerged comparing the two groups in age, gender distribution, baseline symptoms score, and ocular surface parameters. After treatment, both groups demonstrated statistically significant improvements in symptoms scores and ocular surface parameters throughout the follow-up period (Table 2 and Table 3). However, comparing the two groups, there were statistically significant differences in the NIKBUT (*p* = 0.008 and *p* < 0.001, after 15 and 30 days, respectively). Additionally, at three months, the OSDI and SANDE scores (frequency and intensity of symptoms) showed statistically significant improvements in Group 1 (*p* = 0.02, *p* = 0.036, and *p* = 0.032, respectively). Moreover, the bulbar redness score and NIKBUT were significantly improved in Group 1 (*p* < 0.001, respectively). Schirmer I, TMH, and meibographic scores did not demonstrate statistically significant differences between the two groups throughout the follow-up (Table 4). After three months, in Group 1, no patients presented a recurrence of punctal stenosis, whereas in Group 2, three patients presented a proximal canalicular stenosis. Furthermore, no adverse events were reported during the follow-up period in both groups.

## 4. Discussion

To treat acquired punctal and canalicular stenosis, various procedures have been proposed by many authors. The most traditional techniques used by surgeons for this pathology include simple punctal dilation, punctal punching, insertion of a punctal plug, various punctoplasty techniques, insertion of a toughened glass (Lester Jones) tube, external approach DCR, and the application of a stent (such as a vialon cannula) [7,8,9,10,11]. One-, two-, or three-snip punctoplasty alters the tear film drainage system because cuts at the level of the puncta and canaliculi result in the loss of pump function. Hussain et al. described the use of mini-monoka stents, previously employed in canalicular lacerations, in patients with punctal and canalicular stenosis. The study included 73% of patients with punctal stenosis, 72% with canalicular stenosis, and 46% with both conditions coexisting. This surgical approach is less invasive as it is performed under local anesthesia, maintaining the anatomy of the tear drainage system, and the stent was removed after 6 weeks [24,25]. Although 82% of patients showed resolution of epiphora after treatment, there are limited data on the ocular surface.

Another procedure that does not require general anesthesia is the use of balloon catheter dilatation, as presented by Ko et al. [26]. However, this technique has complications, such as extravasation of contrast material, nasal bleeding, and false passages. While immediate resolution of obstruction occurred in approximately 90% and 94% of cases in subjects with complete and partial obstruction, respectively, there were numerous cases of recurrence during follow-up. At six months, only 51% of patients had maintained lacrimal duct patency, decreasing to 43% at 12 months and 40% at 24 months. Additionally, most patients experienced discomfort during the surgical phase.

Despite the various techniques described in the literature, none objectively analyzes quantifiable parameters related to the condition of the ocular surface, which is crucial in the symptomatology of epiphora and inflammation associated with the pathological condition.

A procedure described by Meduri et al. involves inserting a vialon cannula from the inferior canaliculus to the lacrimal sac. This innovative technique uses a stent that is readily available, such as the venous catheter, with the significant advantage that it can be used for tear duct irrigation [10,11].

The use of a venous catheter as an alternative to conventional DCR has several advantages. It is a less invasive technique that ensures tear duct patency, unlike other techniques that may promote fibrosis, resulting in the closure of the entire lacrimal drainage system. Moreover, it allows for the washing of the lacrimal ducts, reducing the risk of post-operative infections [27].

Povidone-iodine (PVI) at 0.6% concentration has been widely used as an antiseptic and disinfectant in eye surgery. Chronic inflammatory stimuli generated by dry eye disease (DED) and Meibomian gland dysfunction (MGD) are still controversial in the pathogenesis of punctal stenosis. However, chronic blepharitis has been implicated in inflammatory and cicatrizing changes. Recent studies have shown that chronic inflammation is a common finding in histological specimens [27].

This study demonstrated the beneficial role of PVI 0.6% in preventing the recurrence of proximal stenosis after surgical treatment in the medium-term follow-up. Patients treated with PVI 0.6% did not present recurrence of stenosis, while in the balanced saline solution (BSS) group, three patients demonstrated punctal stenosis three months after surgical treatment. These results may be related to the antiseptic role of PVI on the microbial flora of the eyelid and ocular surface [28,29]. Bacterial contamination is reported as a significant cause of recurrence of proximal stenosis after surgical treatment, and PVI inhibits the replication of microorganisms by oxidizing the fatty nucleotides and aminoamides of bacterial cell membranes [30,31,32,33].

Moreover, PVI inhibits inflammation of both pathogens and host cells, modulates antioxidant activity and the release of free radicals, reduces the activity of plasmin, inhibits inflammatory mediators such as TNF-a and galactosidase, inhibits the production of metalloproteinases, and modulates the release of pro-inflammatory cytokines. All these mechanisms are involved in the activation and maintenance of inflammation on the ocular surface in patients with DED [13].

Chronic inflammation of ocular surface structures, such as the lid margin, meibomian glands, and conjunctiva, is a major cause of canalicular stenosis. Patients with MGD and chronic blepharitis often present punctal stenosis and epiphora [12,13,14,15]. Tear stasis resulting from canalicular stenosis enhances the persistence of inflammatory mediators on the ocular surface [12,34,35,36]. Resolution of the stenosis facilitates the outflow of the tear film and inflammatory factors, improving the ocular surface. This study demonstrated improvements in dry eye disease (DED) symptoms, tear film stability, and ocular surface parameters. There was a statistically significant difference between the two groups regarding tear film parameters, with better effectiveness in patients receiving PVI 0.6%. Furthermore, a statistically significant improvement in symptoms was demonstrated during the follow-up period (*p* < 0.001) in patients treated with the vialon stent. Consistent with these results, previous studies have confirmed the beneficial role of punctoplasty on symptoms of ocular discomfort using a questionnaire on tear symptoms [37,38]. The beneficial role of PVI 0.6% on tear film stability and ocular surface damage has also been supported by earlier studies.

Limitations of this study include its small sample size, the use of a technique proposed by the research team but not used by other authors in the literature, and introducing significant bias. Prospective studies with a larger sample and comparing others’ techniques are needed to confirm the validity of the data. To our knowledge, this is the first study correlating the application of a venous catheter for proximal stenosis of lacrimal ducts with the prevention of recurrences associated with the irrigation of 0.6% PVI.

## 5. Conclusions

The findings of our study suggest that the application of a venous catheter is an effective treatment for proximal lacrimal duct stenosis. Additionally, irrigation with Povidone-Iodine (PVI) 0.6% appears to reduce inflammation, mitigate the occurrence of fibrosis, and decrease the likelihood of recurrent proximal stenosis. This intervention also demonstrates improvements in tear film stability, reduced damage to epithelial cells, and alleviation of symptoms related to ocular discomfort.

## Figures and Tables

**Table 1:** Clinical characteristics of the study population.

	Group 1	Group 2	*p*-Value
Gender			
Male	4/10 (40%)	7/10 (70%)	0.34
Female	6/10 (60%)	3/10 (30%)	
Age (years)	51.6 ± 21.12	54.9 ± 19.35	0.78
OSDI	17.76 ± 5,58	20.02 ± 9.83	0.21
SANDE (frequency)	48.7 ± 28.39	51.8 ± 21.92	0.32
SANDE (intensity)	47.5 ± 21.92	47 ± 12.74	0.83
SCHIRMER I	26.8 ± 5.79	24.5 ± 5.25	0.56
BULBAR REDNESS	2.51 ± 0.67	2.64 ± 0.64	0.34
TMH	0.39 ± 0.07	0.39 ± 0.04	0.32
NIKBUT	5.82 ± 3.45	5.04 ± 3.74	0.49

**Table 2:** Comparison between pre-operative and post-operative data in Group 1.

	Baseline	15 Days	1 Month	3 Months	*p*-Value
OSDI	17.76 ± 5.58	14.43 ± 4.05	10.9 ± 3.8	8.26 ± 4.06	<0.001
SANDE (frequency)	48.7 ± 28.39	40.7 ± 25.9	29.9 ± 18.87	17 ± 12.4	<0.001
SANDE (intensity)	47.5 ± 21.92	41.6 ± 21.34	32.7 ± 19.2	17.7 ± 12.72	<0.001
SCHIRMER I	26.8 ± 5.79	21 ± 4.83	17 ± 2.58	14.2 ± 1.48	<0.001
BULBAR REDNESS	2.51 ± 0.67	2.21 ± 0.54	1.99 ± 0.49	1.63 ± 0.41	<0.001
TMH	0.39 ± 0.07	0.34 ± 0.05	0.29 ± 0.04	0.26 ± 0.01	<0.001
NIKBUT	5.82 ± 3.45	8.84 ± 3.23	11.95 ± 3.38	13.52 ± 2.36	<0.001

**Table 3:** Comparison between pre-operative and post-operative data in Group 2.

	Baseline	15 Days	1 Month	3 Months	*p*-Value
OSDI	20.02 ± 9.83	18.12 ± 8.91	14.46 ± 5.12	12.83 ± 4.19	<0.001
SANDE (frequency)	51.8 ± 21.92	44 ± 18.44	34.5 ± 13.21	29.9 ± 13.14	<0.001
SANDE (intensity)	47 ± 12.74	39.2 ± 11.7	32.6 ± 10.06	28 ± 11.54	<0.001
SCHIRMER I	24.5 ± 5.25	19.6 ± 3.5	18.3 ± 2.71	15.3 ± 1.7	<0.001
BULBAR REDNESS	2.64 ± 0.64	2.59 ± 0.48	2.53 ± 0.48	2.43 ± 0.35	<0.001
TMH	0.39 ± 0.04	0.31 ± 0.03	0.27 ± 0.03	0.24 ± 0.03	<0.001
NIKBUT	5.04 ± 3.74	4.91 ± 2.69	4.77 ± 2.24	4.82 ± 2.53	<0.001

**Table 4:** Comparison between Group 1 and Group 2 at day 90.

	Group 1	Group 2	*p* Value
OSDI	8.26 ± 3.88	12.83 ± 4.19	0.022
SANDE (frequency)	17 ± 12.4	29.9 ± 13.14	0.036
SANDE (intensity)	17.7 ± 12.72	28 ± 11.54	0.032
SCHIRMER TEST	14.2 ± 1.48	15.3 ± 1.7	0.14
BULBAR REDNESS	1.63 ± 0.41	2.43 ± 0.35	<0.001
TMH	0.26 ± 0.01	0.24 ± 0.03	0.27
NIKBUT	13.52 ± 2.36	4.82 ± 2.53	<0.001

## Data Availability

The original contributions presented in the study are included in the article, further inquiries can be directed to the corresponding author.
